# Supplementary material for: Reinforcement determines the timing dependence of corticostriatal synaptic plasticity in vivo
Source: Nat Commun. 2017 Aug 24;8:334. doi: 10.1038/s41467-017-00394-x (PMC5571189; doi:10.1038/s41467-017-00394-x)
Supplement: Supplementary file 1 — Supplementary Information [file 41467_2017_394_MOESM1_ESM.pdf]

File name: Supplementary Information
Description: Supplementary Figures, Supplementary Methods and Supplementary References

File name: Peer Review File

SUPPLEMENTARY INFORMATION

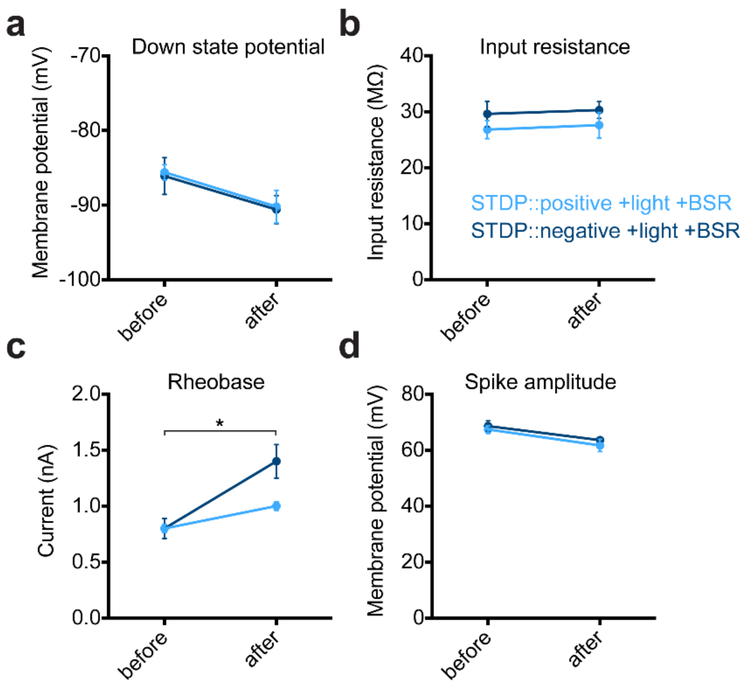

Supplementary Figure 1 | Change in cellular properties due to plasticity protocol. No changes from ‘before’ (following stable impalement) to ‘after’ (following the last PSP measurement of the test phase) time points for positive (n = 7) and negative (n = 5) pairing groups were found in the down state membrane potential **(a)**, the input resistance **(b)**, or the spike amplitude **(d)**. In the negative timing group, the rheobase current **(c)** was significantly different between before and after time points (two-way ANOVA, $F_{1,19} = 30.1$, $P < 0.0001$; Sidak’s multiple comparisons test before-after, STDP::negative, $*P < 0.0001$) suggesting a decrease in intrinsic excitability, although there was no difference for the positive timing group. Increased rheobase was also found, to a similar extent, in the positive STDP with BSR only (no light) group (see Fig. 2h). This group also experienced clear synaptic depression, but did not undergo the cellular processes to do with negative pairings. Since no other groups showed changes in rheobase, it appears that cellular processes associated with synaptic depression in general led to a decrease in excitability.

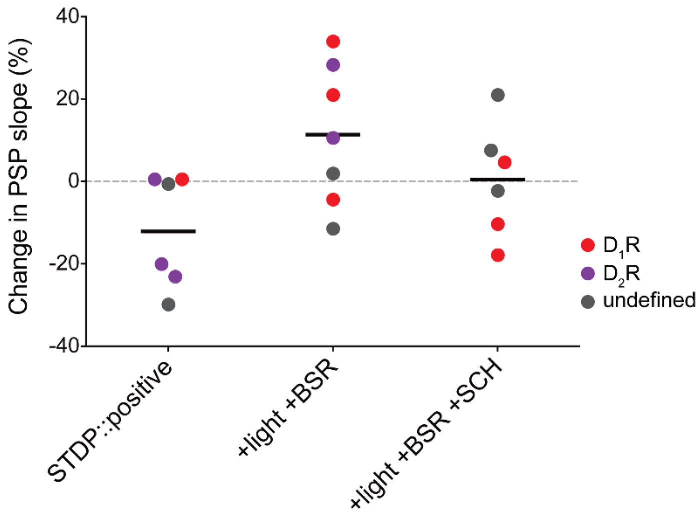

Supplementary Figure 2 | The change in PSP slope due to plasticity protocol in individual cells highlights the variability in the STDP::positive +light +BSR +SCH group. Mean five minute block measures were taken at 20 minutes post protocol. Horizontal lines indicate group mean. Recorded cells are identified as D_1 positive (red), D_2 positive (purple), or as unable to be recovered and defined (see Methods).

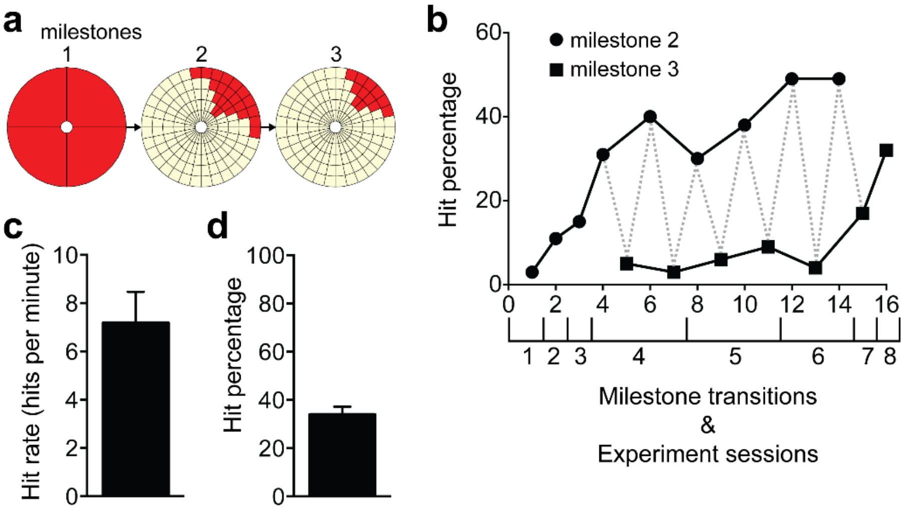

Supplementary Figure 3 | Joystick task training, prior to the switching experiments and manipulations described in Figure 6. (**a**) Training milestones as target areas (in red) to progress through. (**b**) Representative example of learning through the training phase, presented as a hit percentage (hits / [hits + misses]) increase across milestone transitions and experiments. A hit is a movement into the target region and a miss is any other movement of the joystick. Rats progressed from milestone 2 to 3 on achieving performance criteria (hit-to-miss ratio of >0.3 held for >15 minutes, with >80 hits total), and regressed to milestone 2 if they failed to achieve criteria there, as represented by the dashed gray lines. Data for milestone 1 not shown as every movement is a hit. (**c**) Group level learning of milestone 3 is demonstrated by the mean hit rate and mean hit percentage score (**d**) for the final training experiment. The relatively small size of the target region, and the difficulty of the joystick task, are reflected in the final mean performance measures. See Methods for further descriptions of metrics. Data presented as mean ± s.e.m.

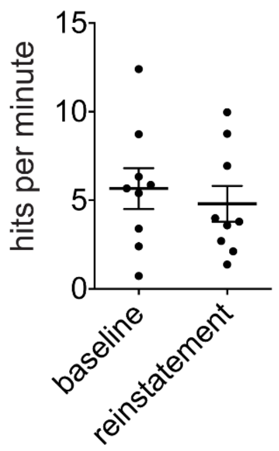

Supplementary Figure 4 | Reinstatement returned performance to baseline. Reinstatement with both reward components (+light +BSR) was performed between manipulation of the reinforcement components by a 15 minute joystick session using the milestone 3 target (Supplementary Fig. S3a), and is demonstrated by a return to baseline hit rate levels (paired, two-tailed *t*-test, $n = 9$, $P = 0.25$). Baseline values are the hit rate during the first 15 minutes of the final training session, which was also with milestone 3. Data presented as mean ± s.e.m.

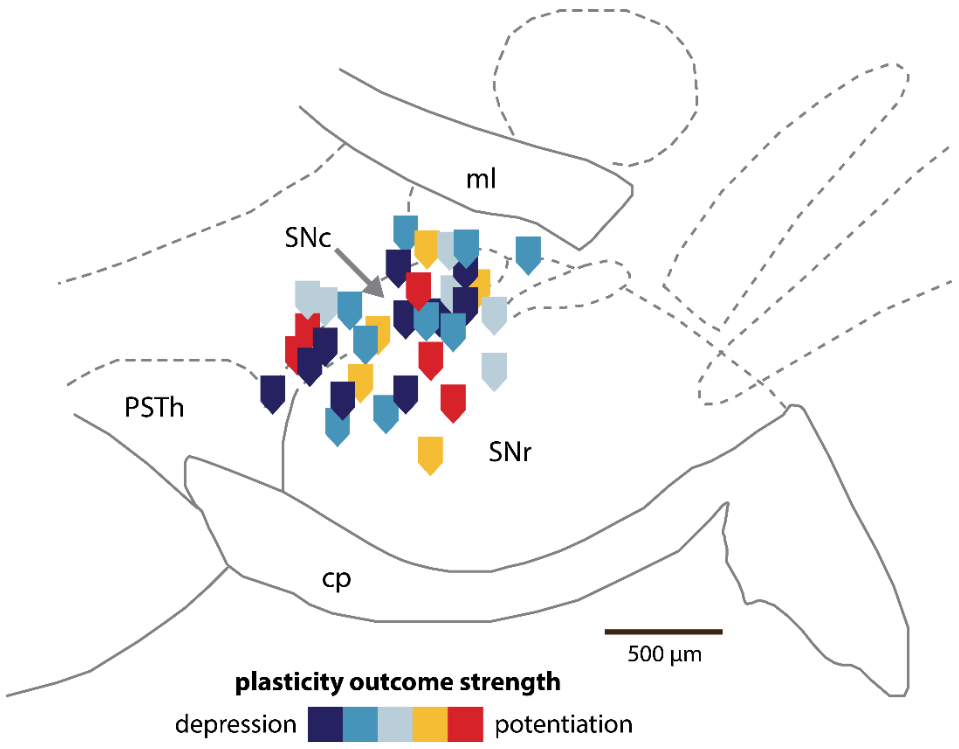

Supplementary Figure 5 | SNc electrode positions of rats included in electrophysiology experiments. A color code indicates the direction and strength of synaptic change. The markers represent the approximate center positions of electrode tips, which in reality measure approximately 0.8 mm in diameter. Additionally, due to their twisted pair structure, the dorsal ventral extent of the conductive area may extend approximately 0.5 mm in either direction. Based on features found at 1.8 mm lateral to Bregma. cp = cerebral peduncle; PSTh = parasubthalamic nucleus; ml = medial lemniscus.

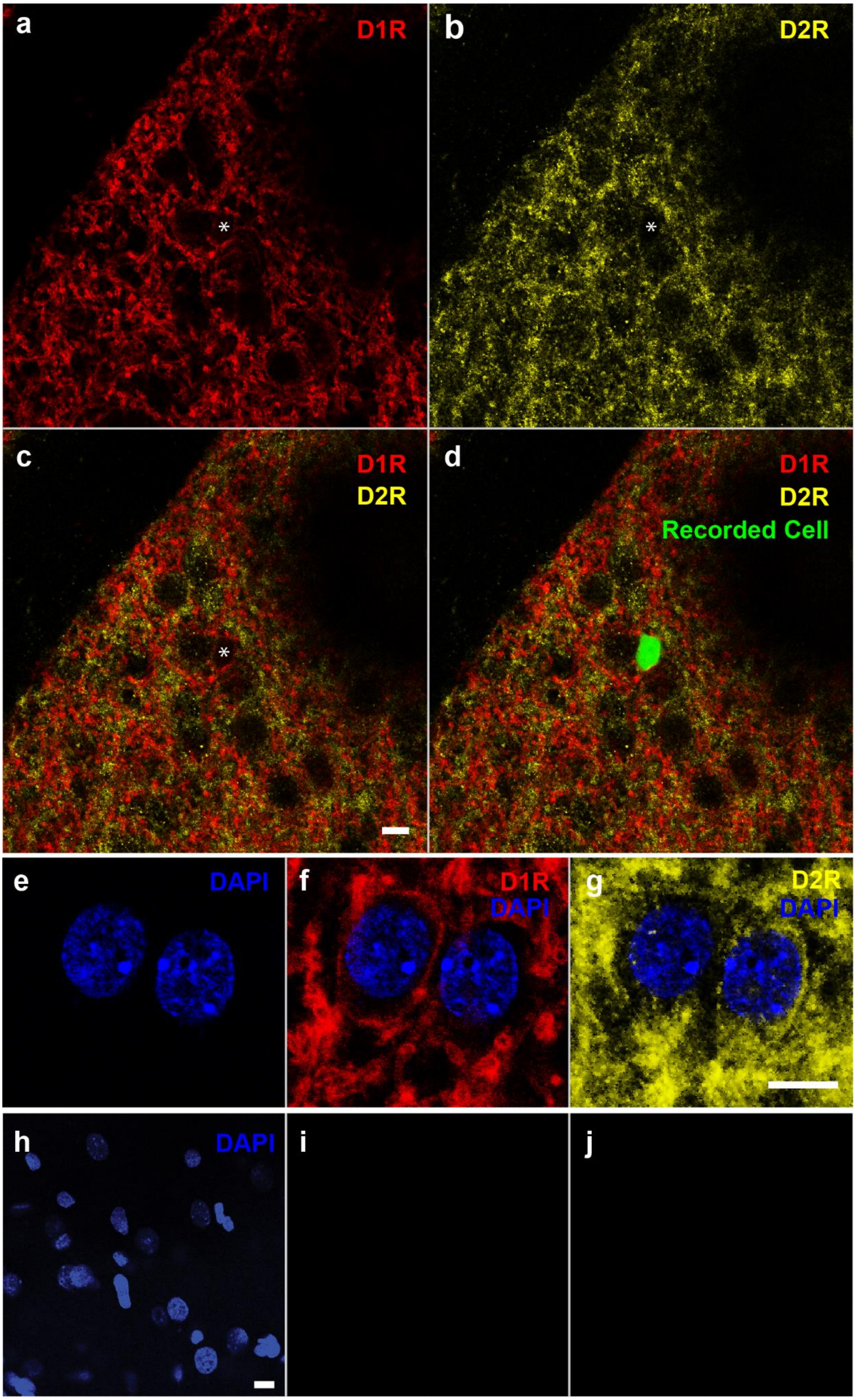
a
D1R
b
D2R
c
D1R
D2R
d
D1R
D2R
Recorded Cell
e
DAPI
f
D1R
DAPI
g
D2R
DAPI
h
DAPI
i
j

Supplementary Figure 6 | Further examples of immunohistochemical staining of striatal neurons using D_1R and D_2R antibodies. (**a**) Additional example of D_1/Cy3 and (**b**) D_2/Cy5 immunostaining, in which the asterisks indicate the recorded cell location. In this example, a perisomal ring is evident in the D_1/Cy3 channel, which was also evident when traversing a stack of images in the Z-axis. (**c**) Combined labelling and (**d**) with the recorded cell visualized with DyLight 488-conjugated streptavidin. (**e**) Higher magnification example, of different cells from a different section, of (**f**) D_1 (Cy3) and (**g**) D_2 (Cy5) positive SPNs, identified by the exclusive presence of a perisomal ring. (**h**) Immunological, secondary only, negative control illustrating DAPI nuclear staining, with (**i**) Cy3 and (**j**) Cy5 staining without primary anti-D_1 and anti-D_2 antibodies. Scale bars = 10 µm.

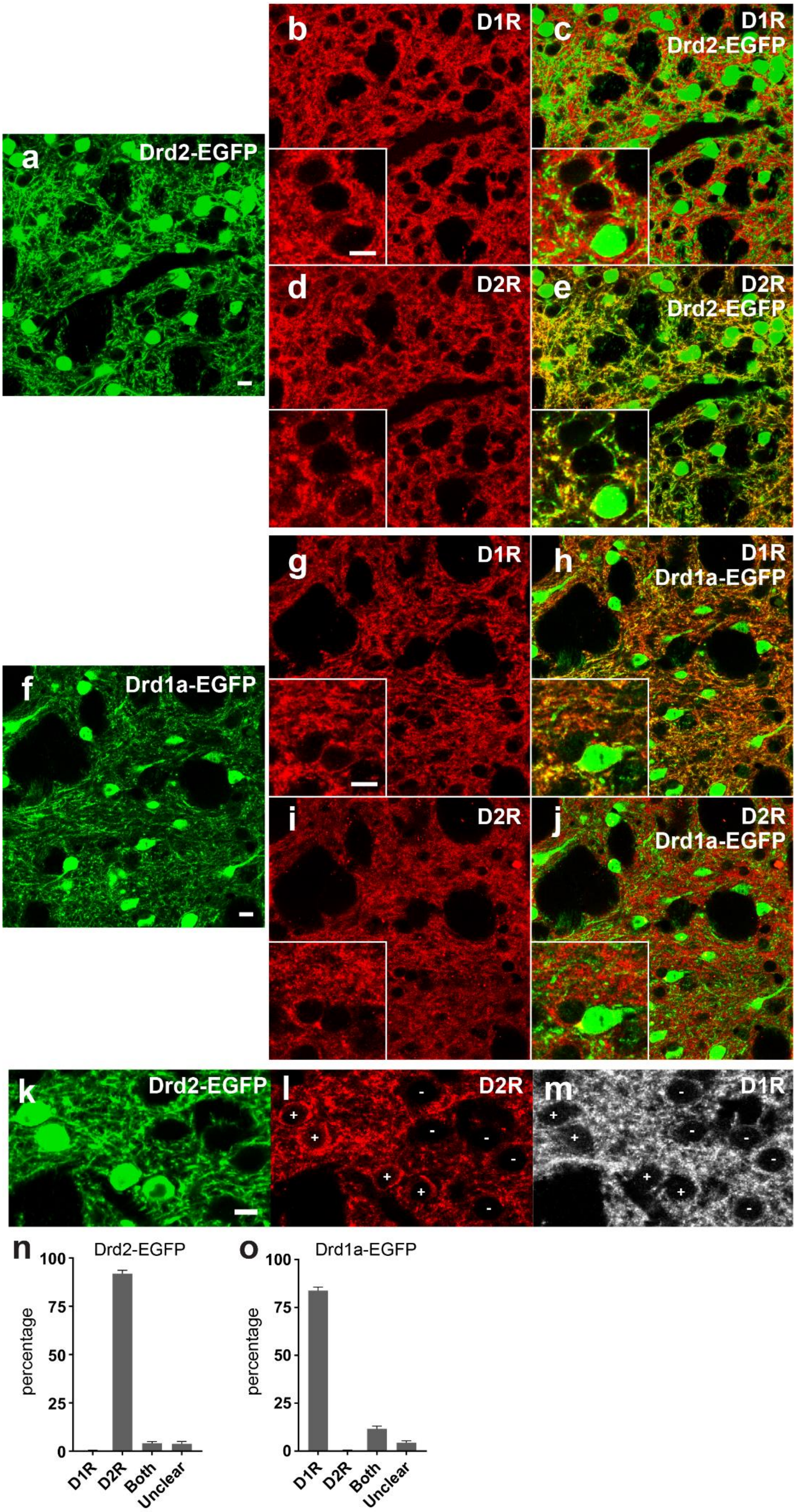
a
Drd2-EGFP
b
D1R
c
D1R
Drd2-EGFP
d
D2R
e
D2R
Drd2-EGFP
f
Drd1a-EGFP
g
D1R
h
D1R
Drd1a-EGFP
i
D2R
j
D2R
Drd1a-EGFP
k
Drd2-EGFP
l
D2R
m
D1R
n
Drd2-EGFP
percentage
100
75
50
25
0
D1R
D2R
Both
Unclear
o
Drd1a-EGFP
percentage
100
75
50
25
0
D1R
D2R
Both
Unclear

Supplementary Figure 7 D_1R and D_2R antibody validation with Drd2-EGFP and Drd1a-EGFP transgenic mice. **(a)** Endogenous EGFP fluorescence in D2R-expressing cells in the dorsolateral striatum. **(b)** D_1R immunofluorescence, and **(d)** D_2R immunofluorescence, labels cells that express D_1R and D_2R respectively. Enlarged insets demonstrate somatic rings and neuropil labeling, and are in approximate register with the adjacent merged labeling. **(c)** Merge of D_1R antibody labeling and Drd2-EGFP labeling indicates very low levels of colocalization. **(e)** Merge of D_2R antibody labeling and Drd2-EGFP labeling indicates a high degree of colocalization. The series **(f-j)** illustrates the same D_1R and D_2R labeling in a Drd1a-EGFP region. In this case, **(h)** the merge of Drd1a-EGP and D_1R indicates high colocalization, and **(j)** the merge of Drd1a-EGFP and D_2R indicates low colocalization. **(k-m)** Further examples of D_2R and D_1R somatic rings used for cell identification. Plus (+) symbols indicate Drd2-EGFP positive cells, and minus symbols (-) negative cells. Scale bars are 10 µm. **(n)** Identified cell counts from a representative sample (n = 351; from 15 dorsolateral striatal regions, from 2 mice) of D_2R-expressing cells in Drd2-EGFP mice. **(o)** Identified cell counts from a representative sample (n = 316; from 15 dorsolateral striatal regions, from 2 mice) of D_1R-expressing cells in Drd1a-EGFP mice. See Supplementary Note for methodological details. Error bars are s.e.m.

Supplementary Methods

Computational model. The two SPNs were modeled using Izhikevich's[1] model:

$$C\dot{v} = k(v - v_{\mathrm{r}})(v - v_{\mathrm{t}}) - u + I \quad \text{if } v \geq v_{\mathrm{peak}} \text{ then}$$
$$\dot{u} = a\{b(v - v_{\mathrm{r}}) - u\} \quad\quad v \leftarrow c, \quad u \leftarrow u + d$$

Where v is the membrane potential, u is the recovery current, I is input current, v_{r} is the resting membrane potential, v_{t} is the instantaneous threshold potential, v_{peak} is spike cutoff value and C is the membrane capacitance. a, b, c, d and k are additional parameters that determine the neuron's behavior. Synaptic strength was governed by the following equation:

$$\dot{S} = k_1 D(t)T(t)E(t) + k_2 T(t)E(t) + k_3 D(t)E(t) + k_4 E(t)$$

Where $S(t)$ is the synaptic strength, $D(t)$ is the dopamine level, $T(t)$ is thalamic activity and $E(t)$ is a synaptic eligibility trace. The constants k_1, k_2, k_3 and k_4 are used to balance the relative strengths of each term. The membrane dynamics of cortical neurons were not directly modeled as their membrane voltage had no effect on our model. To simulate a base level of spontaneous neural activity in the system, the two cortical populations generated random Poisson distributed spike trains at a frequency of 0.5Hz (Poisson parameter $\lambda \cong 2$ for inter-spike time in seconds).

The system of equations for the dopaminergic and thalamic inputs were as follows:

$$C_1 \frac{dD_1}{dt} = I - \frac{D_1}{R_1}$$
$$C_2 \frac{dD_2}{dt} = wD_1 - \frac{dD_2}{R_2}$$

Where D_1 is an intermediate variable without any direct physical meaning, D_2 is the dopaminergic response and I is an input function. The constants C_i, w and R_i are analogous to the membrane capacitance, membrane resistance and input conductance in the standard leaky integrate and fire model. The same system of equations is used

for the thalamic response. The input function I is used to trigger dopaminergic/thalamic response. For the thalamic response, it takes the following form:

$$I(t) = k\delta(t - \tau)$$

Where $\delta(t)$ is the Dirac delta, τ is the time at which thalamic response is triggered and k is a weighting constant. In discrete terms, this equates to an activation pulse a single time-step in duration which triggers the thalamic response. The dopaminergic input function takes the following form:

$$I(t) = k_1\delta(t - \tau_1) + k_2\big(H(t - \tau_2) - H(t - \tau_3)\big)$$

Where $\delta(t)$ is the Dirac delta, $H(t)$ is the Heaviside step function, τ_1 is the time at which a 'conditioned' response is triggered, τ_2 and τ_3 are respectively the start and end times of a simulated BSR-induced dopamine response and k_1 and k_2 are weighting constants.

D_1R and D_2R antibody analysis. As a control for the specificity of the D_1R and D_2R antibodies used, analysis of their labeling was performed in C57Bl/6J–Quackenbush hybrid transgenic mice carrying BAC that express enhanced green fluorescent protein (BAC-EGFP) under the control of D_1R promoter (Drd1a-EGFP) or D_2R promoter (Drd2-EGFP). These mouse lines have been shown to reliably isolate all of the D_1R-positive and D_2R-positive SPNs in the striatum[2,3]. Two Drd2-EGFP, and two Drd1a-EGFP, mice (aged 10-12 weeks) were anaesthetized with isoflurane prior to intracardial perfusion with 4% paraformaldehyde in 0.1 M sodium phosphate buffer (pH 7.4). Brains were extracted and stored in the fixative solution overnight. The brains were then sliced with a vibratome into 40 µM coronal sections through the striatum. Sections were processed with antibodies to label D_1R and D_2R-expressing cells as described in the Methods section, with several modifications. First, an anti-GFP primary antibody (abcam ab13970) and corresponding secondary (Jackson 703-545-155) were added. Second, different secondary antibodies were used for anti-D_1R (Goat anti-Guinea Pig Alexa 647, ThermoFisher A-21450) and anti-D_2R (Goat anti-

Rabbit Alexa 546, ThermoFisher A-11035). Finally, fluorochrome-conjugated streptavidin was not required.

D_1R and D_2R immunofluorescence both exhibited dense staining of the neuropil, with somatic rings around putatively positive cells (Supplementary Fig. 7). Merging D_1R immunolabeling with the Drd2-EGFP signal (Supplementary Fig. 7c) resulted in minimal colocalization, indicating that the D_1R antibody is not also labeling cell bodies and processes of D_2-positive neurons. Conversely, merging D_2R immunolabeling with the Drd2-EGFP signal (Supplementary Fig. 7e) resulted in strong colocalization. The corresponding pattern was also found with the Drd1a-EGFP analysis (Supplementary Fig. 7f-j). This demonstrates that the D_2R and D_1R antibodies are selective for D_2-expressing and D_1-expressing cellular components respectively, as defined by the EGFP labeling.

As described in the Methods section, the presence of somatic rings from anti-D_1R and anti-D_2R labeling allowed for the identification of SPN type. This analysis was performed on striatal slices from Drd2-EGFP and Drd1a-EGFP animals to confirm the validity of the method. For both genotypes, a random selection of 15 220x220 µm regions of the dorsolateral striatum, from both hemispheres of 12 coronal sections, were processed and analyzed. For each region, three images in a Z series of 2-3 µm steps were captured by confocal microscopy, although the cells in only one were analyzed. GFP-positive cells, randomly across regions and between genotypes, were then investigated for their dopamine receptor characterization. The presence of a somatic ring (e.g. Supplementary Fig. 7k-m) from the D_1R or D_2R antibody labeling was used to determine cell type, and reliable identification often required moving through the Z series. If there was not an obvious signal of either cell type the cell was marked as ‘unclear’. Of the 351 Drd2-EGFP cells analyzed, 92% were characterized as exclusively D_2R-expressing, 0% as exclusively D_1R-expressing, 4% as expressing both D_1R and D_2R, and 4% as unclear (Supplementary Fig. 7n). Of the 316 Drd1a-EGFP cells analyzed, 84% were characterized as exclusively D_1R-expressing, 0% as exclusively D_2R-expressing, 11% as expressing both D_1R and D_2R, and 4% as unclear (Supplementary Fig. 7o). These findings suggest that with a given SPN in the dorsolateral striatum an observer is 96% likely to classify it correctly if it is a D_2R-expressing SPN, and 95% likely if it is a D_1R-expressing SPN (single expression +

coexpression of receptors). The proportion of cells expressing both D_1R and D_2R found in the Drd2-EGFP analysis is consistent with a previous report in the dorsal striatum[4].

Supplementary References

1. Izhikevich, E.M. Solving the distal reward problem through linkage of STDP and dopamine signaling. *Cereb. Cortex.* **17**, 2443-2452 (2007).
2. Matamales, M., *et al.* Striatal medium-sized spiny neurons: identification by nuclear staining and study of neuronal subpopulations in BAC transgenic mice. *PLoS One* **4**, e4770 (2009).
3. Valjent, E., Bertran-Gonzalez, J., Herve, D., Fisone, G. & Girault, J.A. Looking BAC at striatal signaling: cell-specific analysis in new transgenic mice. *Trends Neurosci.* **32**, 538-547 (2009).
4. Bertran-Gonzalez, J., *et al.* Opposing patterns of signaling activation in dopamine D1 and D2 receptor-expressing striatal neurons in response to cocaine and haloperidol. *J. Neurosci.* **28**, 5671-5685 (2008).
